# Development and Application of Genomic Resources in an Endangered Palaeoendemic Tree, *Parrotia subaequalis* (Hamamelidaceae) From Eastern China

**DOI:** 10.3389/fpls.2018.00246

**Published:** 2018-03-01

**Authors:** Yun-Yan Zhang, En Shi, Zhao-Ping Yang, Qi-Fang Geng, Ying-Xiong Qiu, Zhong-Sheng Wang

**Affiliations:** ^1^College of Life Sciences, Nanjing University, Nanjing, China; ^2^Key Laboratory of Conservation Biology for Endangered Wildlife of the Ministry of Education, College of Life Sciences, Zhejiang University, Hangzhou, China; ^3^College of Life Sciences, Tarim University, Alaer, China; ^4^Asian Natural Environmental Science Center, The University of Tokyo, Tokyo, Japan

**Keywords:** *Parrotia subaequalis*, palaeoendemic tree, chloroplast genome, microsatellites, fragmentation, conservation genetics

## Abstract

*Parrotia subaequalis* is an endangered palaeoendemic tree from disjunct montane sites in eastern China. Due to the lack of effective genomic resources, the genetic diversity and population structure of this endangered species are not clearly understood. In this study, we conducted paired-end shotgun sequencing (2 × 125 bp) of genomic DNA for two individuals of *P. subaequalis* on the Illumina HiSeq platform. Based on the resulting sequences, we have successfully assembled the complete chloroplast genome of *P. subaequalis*, as well as identified the polymorphic chloroplast microsatellites (cpSSRs), nuclear microsatellites (nSSRs) and mutational hotspots of chloroplast. Ten polymorphic cpSSR loci and 12 polymorphic nSSR loci were used to genotype 96 individuals of *P. subaequalis* from six populations to estimate genetic diversity and population structure. Our results revealed that *P. subaequalis* exhibited abundant genetic diversity (e.g., cpSSRs: *H*cp = 0.862; nSSRs: *H*_T_ = 0.559) and high genetic differentiation (e.g., cpSSRs: *R*_ST_ = 0.652; nSSRs: *R*_ST_ = 0.331), and characterized by a low pollen-to-seed migration ratio (*r* ≈ 1.78). These genetic patterns are attributable to its long evolutionary histories and low levels of contemporary inter-population gene flow by pollen and seed. In addition, lack of isolation-by-distance pattern and strong population genetic structuring in both marker systems, suggests that long-term isolation and/or habitat fragmentation as well as genetic drift may have also contributed to the geographic differentiation of *P. subaequalis*. Therefore, long-term habitat protection is the most important methods to prevent further loss of genetic variation and a decrease in effective population size. Furthermore, both cpSSRs and nSSRs revealed that *P. subaequalis* populations consisted of three genetic clusters, which should be considered as separated conservation units.

## Introduction

The genus *Parrotia* C. A. Mey. (Hamamelidaceae) contains just two extant species, *Parrotia persica* (DC.) C. A. Mey. and *P. subaequalis* (Hung T. Chang) R.M. Hao & H.T. Wei (Dirr et al., 1998; Wang and Xie, 2004; Andrews, 2007; Sefidi et al., 2014; Angiosperm Phylogeny Group, 2016). The former is native to the Alborz Mountain of northern Iran, whereas the latter is restricted to eastern China (Li and Tredici, 2008; Sefidi et al., 2011). Due to their unique exfoliating bark, obovate leaves with gorgeous autumn color, and beautiful apetalous flowers, both species are cultivated as ornamental trees in North America, Europe and East Asia (Nicholson, 1989; Li and Zhang, 2015). *P. subaequalis* differs from *P. persica* in its lanceolate stipules and sepals fused into a shallow saucer-shaped calyx (Hao et al., 1996; Li and Tredici, 2008). The two species of *Parrotia* were estimated to diverge at around 7.5 Mya (Li and Tredici, 2008). This speciation event is attributable to range fragmentation of the widespread Tertiary vegetation, as colder and more arid climates developed shortly after the mid-Miocene (Yin and Harrison, 2000; Sun et al., 2005).

*Parrotia subaequalis*, the focal species of the present study, is a diploid (2*n* = 2*x* = 24), deciduous tree (up to 6–8 m tall) with bisexual and wind-pollinated flowers that turn into woody capsules, dispersed by gravity and water (Li et al., 1999; Li and Tredici, 2008). *P. subaequalis* grows mainly in a mixed evergreen and deciduous broadleaved forest (*c*.100–700 m above sea level) on the hillsides and along canyons in subtropical China, where it is presently found in only about 8 known populations from disjunct/montane sites in Anhui, Jiangsu, Zhejiang and Henan provinces of eastern China (Hu et al., 2011; Li and Zhang, 2015). Because of its extremely restricted distribution and very few extant populations, *P. subaequalis* is classified as “extremely endangered” by the IUCN (International Union for Conservation of Nature; Bhandari, 2012) and Chinese Plant Red Book (Grade I Key protected Wild Plant; Wang and Xie, 2004). The limited geographic range of this species can be ascribed to the species alternate-year fruit production, serious habitat destruction, and increasing anthropogenic disturbance in the form of timber harvesting and clearing forest land for agriculture (Li and Tredici, 2008; Li and Zhang, 2015). Previous studies have focused primarily on germplasm investigation, taxonomy, systematics, population ecology, cultivation and ecophysiology (Li et al., 1999; Yan et al., 2008; Yao et al., 2010; Zhong et al., 2014). However, although genetic information is crucial for conservation and management strategies of a rare species (Frankham, 2010; Nybom et al., 2014), only very few studies have been conducted to assess genetic diversity and population structure of *P. subaequalis*. For example, the spatial patterns of genetic variation of *P. subaequalis* were surveyed for five natural populations using inter-simple sequence repeats (ISSRs) (Geng et al., 2015). The results revealed high genetic differentiation (*G*_ST_ = 0.46) and low within-population diversity 0.11 for Nei's gene diversity. However, the dominant nature of ISSR markers precludes estimating small-scale spatial genetic structure and inbreeding coefficient *F*_IS_ as well as inferring evolutionary/demographic history which are necessary to guide management practices aiming at avoiding inbreeding and assessing future risk of erosion of diversity (Turlure et al., 2014). By contrast, the highly variable co-dominant nuclear microsatellite (SSR) markers are suitable for inferring relatively recent population genetic events and detecting the possible inbreeding (Perdereau et al., 2014). In addition, without the confounding effect of biparental inheritance, the maternally inherited chloroplast (cp) DNA markers could provide information on the migration routes and location of refugia of species (Avise, 2000).

To enable further population genetic and phylogeographic studies in *P. subaequalis*, we developed polymorphic nSSRs and cpSSRs markers for this species. Compared with traditional SSR development using random enrichment strategies, new methods to identify SSRs from next-generation sequencing (NGS) datasets are less expensive and more efficient (Squirrell et al., 2003; Li et al., 2015, 2017; Stoll et al., 2017; Yang et al., 2017). Here, we performed low-coverage whole genome shotgun sequencing for two *P. subaequalis* individuals from the northern- and southernmost population on the Illumina HiSeq 2500 platform. The sequencing data of *P. subaequalis* generated enough reads to assemble the entire chloroplast genome, from which maternally inherited markers, including highly variable regions and cpSSR loci can be developed. Moreover, we identified nSSR loci by searching in scaffolds for SSR motifs from the genomic sequences. The candidate loci were chosen for designing primer pairs, which were further tested for PCR amplification followed by screening for polymorphisms. Finally, we employed 10 cpSSRs and 12 nSSRs to investigate the genetic variation and population structure in six populations sampled throughout most of its natural range. The results here will provide useful resources for further studies on the relative role of historical and contemporary evolutionary processes in determining the contemporary genetic diversity of this Tertiary relict species, and lay a scientific foundation for designing conservation strategies.

## Materials and methods

### Plant samples and DNA extraction

**Figure 1:**
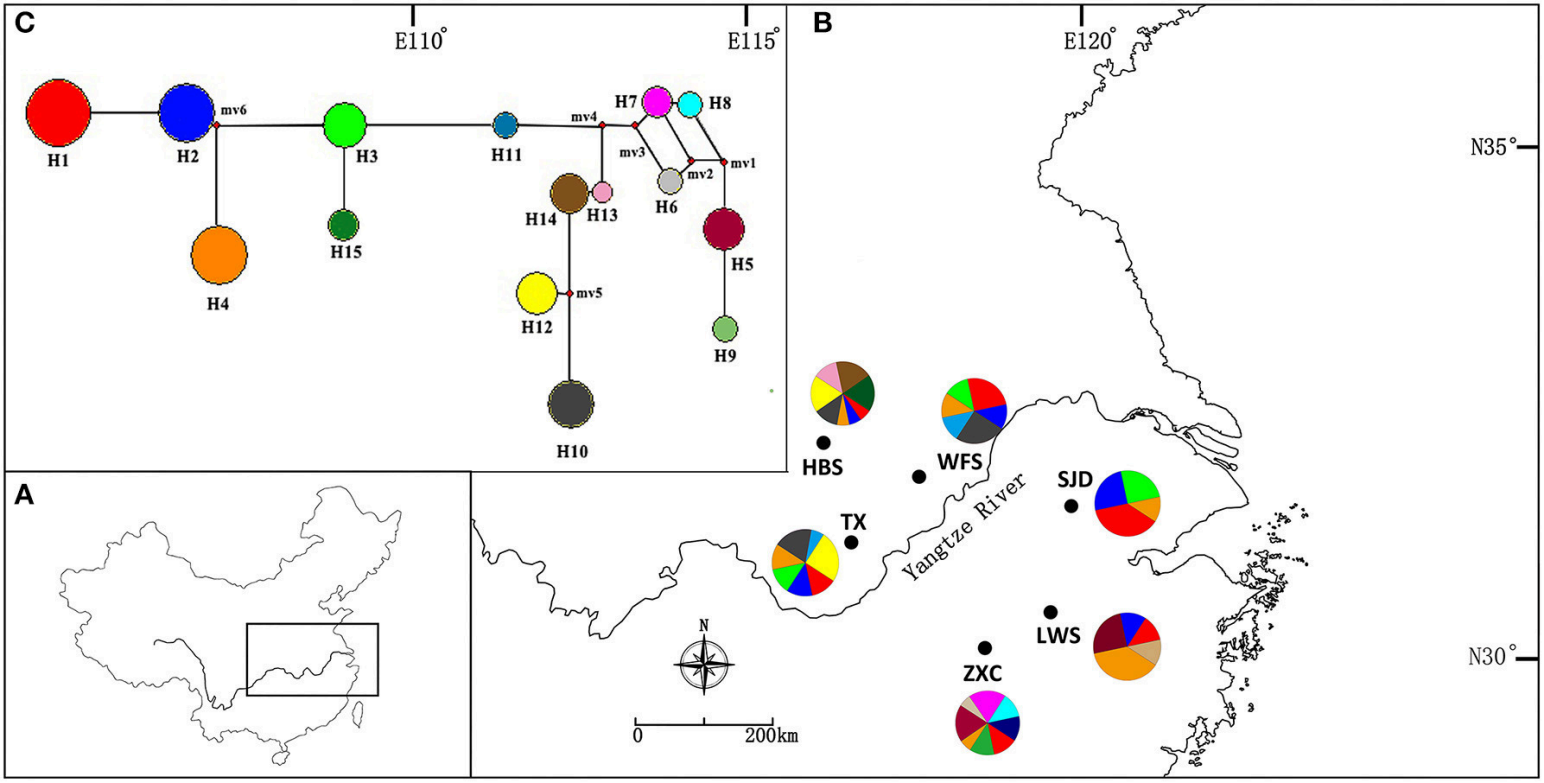
**(A)** The distribution range of *Parrotia subaequalis* in China. **(B)** Location of the six extant natural populations and geographic distribution of 15 cpSSR haplotypes (H1-H15). The radius of the pie charts is proportional to the number of individuals sampled. **(C)** Median-joining network showing the relationship of haplotypes. The haplotypes are indicated by circles and the colors correspond with the color of the haplotypes in all populations, and small red circles show median vectors. The size of each pie chart is proportional to the frequency of corresponding haplotype.

The fresh leaves of two individuals were collected from the population TX in Anhui Province and the population SJD in Jiangsu Province (China) (Table 1), respectively. They were chosen to represent the northern- and southernmost distribution of *P. subaequalis*. To evaluate and validate the polymorphism of both the nSSRs and cpSSRs developed from our sequencing data, leaf material of *P. subaequalis* was collected from single individuals in six populations from across the distribution range of the species in eastern China. From each population, 16 individuals were sampled at least 10 m apart to minimize the likelihood of sampling clones, resulting in a total of 96 individuals (Figure 1, Table 1). The polymorphic nSSRs and cpSSRs were further tested for transferability to five related species in Hamamelidaceae, i.e., *Parrotia persica, Parrotiopsis jacquemontana, Sycopsis sinensis, Distylium racemosum, Hamamelis virginiana* (Table 1). For each species, three accessions were used. Collected leaf material was dried with silica gel for further DNA extraction. Representative voucher specimens of all populations sampled were deposited in the Herbarium of Zhejiang University (HZU). Total genomic DNA was extracted from the silica-dried leaves with DNA Plantzol Reagent (Invitrogen) following the manufacturer's protocol. The quality of the DNA was determined by band intensity and integrity from electrophoresis on a 0.8% agarose gel stained with 1 × GelRed (Biotium). Besides, the concentration of DNA was tested by an Aglient BioAnalyzer 2100 (Agilent Technologies).

**Table 1:** Voucher information and geographic characteristics of 6 *Parrotia subaequalis* populations and five related species from Hamamelidaceae.

**Species**	**Population code**	**Voucher specimens^a^**	**Collection locality**	**Geographic coordinates**	**Altitude (m)**	***n***
*Parrotia subaequalis*	SJD	Yunyan Zhang, ZYY16082401	Shan Juan Cave, Jiangsu Province, China	31.5806N, 119.8004E	312-485	16
*P. subaequalis*	LWS	Yunyan Zhang, ZYY16090704	Mt. Longwang, Zhejiang Province, China	30.2454N, 119.2484E	876-1238	16
*P. subaequalis*	ZXC	Yunyan Zhang, ZYY16090805	Zhuxian Village, Anhui Province, China	30.1259N, 118.5405E	718	16
*P. subaequalis*	WFS	Yunyan Zhang, ZYY16082903	Mt. Wangfo, Anhui Province, China	32.1492N, 117.4469E	461-687	16
*P. subaequalis*	TX	Yunyan Zhang, ZYY16083002	Mt. Tianxia, Anhui Province, China	30.8918N, 116.3617E	417-522	16
*P. subaequalis*	HBS	Yunyan Zhang, ZYY16091806	Mt. Huangbo, Henan Province, China	33.4340N, 116.0471E	682-899	16
*Parrotia persica*	–	Pan Li, LP174414	Chenshan Botanical Garden, Shanghai, China	31.4545N, 121.1038E	17	3
*Parrotiopsis jacquemontana*	–	Pan Li, LP174412	Zurich University Botanical Gardens, Switzerland	47.2229N, 8.3255E	435	3
*Sycopsis sinensis*	–	Pan Li, LP172890	Hangzhou Botanical Garden, Zhejiang Province, China	30.1514N, 120.7171E	18	3
*Distylium racemosum*	–	Pan Li, LP172887	Hangzhou Botanical Garden, Zhejiang Province, China	30.1514N, 120.7171E	20	3
*Hamamelis virginiana*	–	Pan Li, CB08834	Mt. Wujia Forest Farm, Hubei Province, China	31.0426N, 116.1534E	931	3

*n, number of individuals sampled*;

a *Vouchers were deposited in the Herbarium of Zhejiang University (HZU), Hangzhou, Zhejiang Province, China*.

### Illumina paired-end sequencing and chloroplast DNA genome assembly, validation, and annotation

Two next-generation sequencing (NGS) DNA libraries were prepared using the low-coverage shotgun sequencing of the whole genome of two *P. subaequalis* individuals. Firstly, a total of 3 μg of the purified genomic DNA was used for each library construction. Short-insert (500 bp) paired-end libraries were generated in accordance with the Illumina standard protocol. Two libraries with different indexes were then pooled together and sequenced in one lane of HiSeq 2500 platform (Illumina Inc., San Diego, California, USA) at Beijing Genomics Institute (Shenzhen, China). For each individual, about 2.5 Gb of raw data were obtained with pair-end 125 bp read length.

Paired-end reads of *P. subaequalis* were assembled into whole chloroplast genomes using a combination of both reference guide and *de novo* assembly approaches (Cronn et al., 2008). First, to obtain the high-quality clean data, all raw reads of two accessions were filtered by removing adapter sequences and low-quality reads (*Q* < 20, 0.01 probability error) using CLC-quality trim program clc assembly cell package (http://www.clcbio.com/products/clc-assembly-cell/). Then, for the reference-based mapping, we aligned the trimmed reads to the reference chloroplast genome of *Liquidambar formosana* Hance (**Genbank ID: KC588388**) using bowtie software (Langmead et al., 2009). *De novo* assembly of reads were then conducted using clc genomics workbench v8 (http://www.clcbio.com) under the following parameters: minimum contig length = 200 bp, bubble size = 98 bp, deletion and insertion costs = 3, mismatch cost = 2, length fraction = 0.9, and similarity fraction = 0.8. All the assembled contigs were aligned and ordered with respect to the reference cp genome of *L. formosana* through blast. Lastly, the top-quality reads were again mapped to the draft cp genomes to yield the complete cp genomes of two *P. subaequalis* individuals that were visualized in geneious v10.0.5 (http://www.geneious.com). To confirm accuracy, the four junctions between LSC/IRs and SSC/IRs of the two cp genomes of *P. subaequalis* were validated via specific primers as described previously (Dong et al., 2013) and PCR-based conventional Sanger sequencing.

Gene annotation of the two cp genomes of *P. subaequalis* was conducted using the program dual organellar genome annotator (dogma; Wyman et al., 2004), and checked manually for gene start and stop codons. All protein-coding genes, rRNAs and tRNAs were identified by using the plastid/bacterial genetic code. Intron/exon boundaries were corrected by comparison with homologous genes from *L. formosana* using mafft v7 (Katoh and Standley, 2013). Furthermore, tRNAscan-SE (Schattner et al., 2005) was employed to verify the tRNA genes. Finally, the whole chloroplast genome maps were generated using Organellar Genome draw (Lohse et al., 2013), with subsequent manual editing. Codon usage and relative synonymous codon usage (RSCU, Sharp and Li, 1987) value were estimated for all protein-coding genes via the program codonw v1.4.2 (http://codonw.sourceforge.net/).

### Identification of microsatellite loci and highly variable regions

To exploit simple sequence repeats (SSRs) within the cp genomes, we applied the misa perl script (Thiel et al., 2003) with the following repeat thresholds settings: 10 repeat units for mononucleotide SSRs, 5 for dinucleotide SSRs, 4 for trinucleotide SSRs, and 3 for tetra-, penta-, and hexa-nucleotide SSRs. Additionally, in order to identify the nuclear genome database, we firstly removing the contigs used for the assembly of the two cp genomes and then the mitochondria-contained contigs were further removed from the assembled genome database using the mitochondrial genomes of *Heuchera parviflora* var. *saurensis* (**Genbank ID: KR559021**) and *Vitis vinifera* (**Genbank ID: FM179380**) as references. After filtering the cp- and mitochondria-contained contigs, potential polymorphic nSSRs were identified using CandiSSR (Xia et al., 2016) from the remaining scaffolds of the assembled nuclear genome database.

Both coding regions and noncoding regions with total number of mutation (Eta) > 0 and an aligned length > 200 bp were extracted to explore the divergence hotspot regions in *P. subaequalis* cp genome using mafft v7. The sequence divergence for a specific region between two cp genomes was quantified as the nucleotide variability (Pi) and estimated using dnasp v5.10 (Librado and Rozas, 2009).

### SSR primer design and polymorphism assessment

Nuclear and cp SSR primer pairs were designed according to the flanking sequences of the SSR loci using Primer3 package (http://sourceforge.net/projects/primer3/files/primer3/) with the following parameters: 18–25 base pairs (bp) in length, PCR product size of 100–300 bp, annealing temperature of 50°-65°C, and a GC content of 40–60%. After that, according to our potential polymorphic SSRs selection principles, we finally selected 20 chloroplast and 30 nuclear candidates of polymorphic SSRs primer pairs to do the further trials (Tables S5, S7). Potential polymorphic cpSSRs identification criteria were as follows: (1) polymorphic cpSSRs possessed the same repeat units with the different number of repeat units; (2) for the cp mononucleotide repeats, more than ten repeat numbers were far more likely to be polymorphic cpSSRs; whereas for the di-, tri-, or tetranucleotide repeats comprising four or more repeat units is likely to represent a variable locus (Jakobsson et al., 2007; Ebert and Peakall, 2009); (3) polymorphic cpSSRs were located in the homologous non-coding regions. Besides, according to the results of CandiSSR, specific potential polymorphic nSSRs selected standards were as follows: (1) the nSSRs which the missing rate value were equal to zero and the transferability/similarity value were equal to one were chosen; (2) every type of nucleotide repeats motif, such as the di-, tri-, tetra-, penta-, or hexanucleotide repeats were selected; (3) on the premise of the above two principles, for each type of motif, we just chose a few of them to have a further polymorphism test.

Primer pairs were initially screened for amplification success and effectiveness using one randomly selected individual. The polymorphism of each locus was performed then by selecting 12 individuals from six representative geographically distant populations (two individuals were chosen from each population at random). PCR amplifications were performed on a GeneAmp9700 DNA Thermal Cycler (Perkin-Elmer, Waltham, Massachusetts, USA) following the standard protocol of the AmpliTaq Gold 360 Master PCR kit (Thermofisher Biotech Company, Applied Biosystems, Foster City, California, USA) in a final volume of 15 μL, which contained 1 μL of template DNA, 7.5 μL AmpliTaq Gold 360 Master Mix (Thermofisher Biotech Company, Applied Biosystems, Foster City, California, USA), 5.5 μL of deionized water, and 0.5 μL of forward and reverse primers (10 μM). And the procedure of PCR was 5 min initial denaturation at 95°C, 35 cycles of 45 s at 95°C, 30 s annealing at optimal primer temperature (Tables S6, S8) and 30 s synthesis at 72°C, followed by a final 10-min extension step at 72° and a 4°C holding temperature. PCR products were separated on 6.0% (w/v) denaturing polyacrylamide gels (PAGE) and subsequently visualized by silver staining. After the identification of the polymorphism, we used 96 individuals from six representative geographically distant populations to further amplify and character the polymorphic SSR loci. The forward primer of each of the polymorphic primer pairs was labeled with a fluorescent dye (FAM, ROX, HEX, or TAMRA; Tables S6, S8), and SSR loci were amplified using the PCR conditions described above.

Fragment lengths of PCR products were analyzed on an ABI 3730XL DNA Analyzer (Applied Biosystems, Foster City, California, USA) with GeneScan LIZ 500 as an internal reference (Applied Biosystems). Electrophoresis peaks scoring and alleles identification were assayed by using GeneMarker v2.2.0 (SoftGenetics, State College, Pennsylvania, USA). All primer sequences obtained from this study were submitted to GenBank (Tables S6, S8). In addition, transferability among the other five Hamamelidaceae species was assessed using the same PCR conditions described above.

### Population structure and differentiation at chloroplast and nuclear SSR loci

Each cpSSR was considered a locus at a specific site and length variants were considered as alleles. Different alleles at each of the cpSSR loci in each individual were combined into one haplotype because the cp genome behaves as a single non-recombining locus. For each polymorphic locus, we obtained the number of alleles (*N*_A_) and unbiased haploid diversity index (*h*_d_) using the program GenAlEx v6.41 (Peakall and Smouse, 2006). For each population, we computed haplotypic diversity (*H*cp), the number of effective alleles (*N*_E_) using program contrib v1.02 (Petit et al., 1998). Two coefficients of population differentiation, *G*_ST_ and *R*_ST_, were analyzed using the program permut & cpssr v2.0 (Pons and Petit, 1996). *G*_ST_ is only based on haplotype frequency, while *R*_ST_ takes into account both haplotype frequencies and haplotype similarities. A significantly higher *R*_ST_ than *G*_ST_ values (1,000 permutations; *P* < 0.05) implies that genealogically closely related haplotypes are geographically close to each other, indicating the presence of phylogeographical structure (Pons and Petit, 1996). A network of relationship between cpDNA haplotypes was constructed using a median-joining network (MJN, Bandelt et al., 1999) approach implemented in network v5.0.0.3 (http://www.fluxus-engineering.com/). Analysis of molecular variance (AMOVA) was performed to partition the total genetic variance among populations using the arlequin v3.11 (Excoffier et al., 2007). Significance tests were made after 1,000 permutations.

For each nSSR locus, the number of alleles (*N*_A_), allelic richness (*A*_R_), expected heterozygosity (*H*_E_), observed heterozygosity (*H*o), total genetic diversity (*H*_T_), average genetic diversity within populations (*H*_S_) and polymorphism information content (PIC) were calculated by using cervus v2.0 (Kalinowski et al., 2007) and fstat v2.9.3.2 (Goudet, 2001). Deviation from Hardy-Weinberg equilibrium (HWE) for each primer pair was tested using a Markov chain (dememorization 1,000, 100 batches, 1,000 iterations per batch) in genepop v4.0.7 (Rousset, 2008). The frequency of null alleles and their bias on genetic diversity were evaluated based on the expectation maximization method implemented in freena (Chapuis and Estoup, 2006). Differentiation between populations (*R*_ST_; Slatkin, 1995) under a stepwise mutation model (SMM; Kimura and Ohta, 1978) was computed in arlequin v3.11. All nSSR samples were assigned to genetic clusters (*K*) by using structure v2.3.4 (Pritchard et al., 2000), assuming the admixture model with independent allele frequencies. This program was run 10 times for each *K* value ranging from 1 to 6 (the number of sampled localities) to determine the maximum value of posterior probability of the data [ln*p*(D)] (Pritchard et al., 2000). Each run was performed using a burn-in period of 10^4^ and a run length of 10^5^ iterations. In addition, we estimated a parameter (Δ*K*) that corresponds to the change of ln*p*(D) between consecutive *K* values (Evanno et al., 2005). The most likely number of cluster (*K*) was identified as the one that maximized ln*p*(D) and/or Δ*K*. Analysis of molecular variance (AMOVA) was performed to partition the total genetic variance among populations using the arlequin v3.11.

We tested for a correlation between pairwise population genetic distance (*R*_ST_) and geographic distance among sampling locations (isolation-by-distance effect) using a Mantel test (Mantel, 1967) in IBDWS (Isolation by Distance Web Service) v3.21 (Jensen et al., 2005) with 10,000 permutations to determine the significance of IBD pattern. Additionally, we calculated the pollen/seed migration ratio (*r*) using a modified form of an equation of Ennos (1994) and used *R*_ST_ values as estimators of population differentiation following Petit et al. (2005): *r* = mp/ms = [(1/*R*_ST(n)_-1)−2(1/*R*_ST(c)_-1)]/(1/*R*_ST(c)_-1), where mp and ms correspond to the pollen migration rate and the seed migration ratio, *R*_ST(n)_ is the nuclear *R*_ST_ and *R*_ST(cp)_ is the cytoplasmic *R*_ST_.

## Results

### Chloroplast DNA genome organization and structural features

After filtering the low-quality reads and adaptor sequences, 34,688,769 and 25,632,935 clean reads (of 125 bp length) were produced for *P. subaequalis* (TX) and *P. subaequalis* (SJD), respectively. The *de novo* assembly generated 359,167 contigs with an N_50_ length of 554 bp for *P. subaequalis* (TX) and 274,639 contigs with an N_50_ length of 580 bp for *P. subaequalis* (SJD) (Table 2). Subsequently, based on total alignment score and percentage sequence identity, three to four initial contigs were identified with greatest similarity to the reference cp genome of *L. formosana*. They were combined to generate each draft chloroplast genome, with no gaps or missing nucleotids (Ns). Four junction regions in each chloroplast genome were validated using PCR-based sequencing (Table S1). These sequences were found to be identical with the assembled genomes, confirming the accuracy of our genome sequencing and assembly.

**Table 2:** The basic characteristics of two chloroplast genomes of *Parrotia subaequalis*.

**Characteristics**	***P. subaequalis* (TX)**	***P. subaequalis* (SJD)**
GenBank accession number	MG252374	MG334121
Clean reads	34,688,769	25,632,935
Average read length (bp)	125	125
Number of contigs	359,167	274,639
N_50_ length of contigs (bp)	554	580
Total cpDNA size (bp)	159,280	159,324
LSC length	87,927	87,968
SSC length	19,031	18,932
IR length	26,211	26,212
Total GC content (%)	38.0	38.0
LSC	36.1	36.1
SSC	32.4	32.4
IR	43.1	43.1
Total number of genes	133	133
Protein-coding genes	81	81
rRNA genes	4	4
tRNA genes	30	30
Duplicated genes	18	18

**Figure 2:**
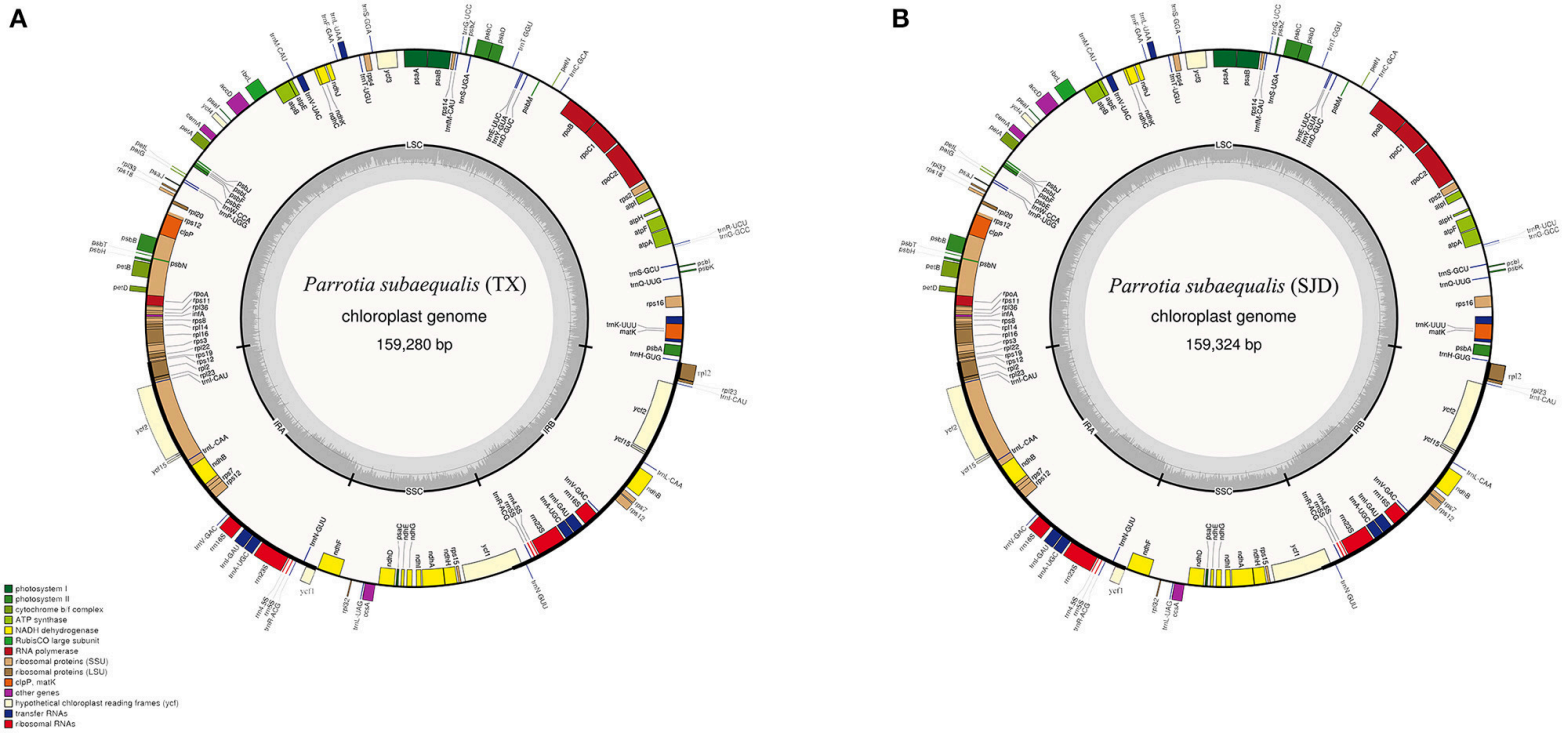
Gene maps of the two *Parrotia subaequalis* chloroplast genomes. **(A)** *Parrotia subaequalis* (TX); **(B)** *Parrotia subaequalis* (SJD). Genes shown on the outside of the circle are transcribed clockwise, and genes inside are transcribed counter-clockwise. Genes belonging to different functional groups are color-coded. The darker gray in the inner corresponds to GC content, and the lighter gray corresponds to AT content.

The whole cp genome sequences of two *P. subaequalis* were 159,280 bp (**TX; GenBank: MG252374**) and 159,324 bp in length (**SJD; GenBank: MG334121**) (Table 2, Figure 2). The cp genomes of *P. subaequalis* exhibited a typical quadripartite structure, consisting of a pair of inverted repeats (IRs) with a length of 26,211 in TX and 26,212 bp in SJD, separated by one large single-copy (LSC) region of 87,927 bp in TX and 87,968 bp in SJD and a small single-copy (SSC) region of 19,031 bp in TX and 18,932 bp in SJD. The gene map is shown in Figure 2. The overall GC content was 38.0%, whereas the GC content in the LSC, SSC, and IR regions were 36.1, 32.4, and 43.1%, respectively (Table 2). Gene content and arrangement were identical in both cp genomes. There were 133 predicted functional genes in both genomes, of which 115 were unique and 18 were duplicated in the IR regions. Among the 115 unique genes, there were 81 protein-coding genes, 30 tRNA genes and 4 rRNA genes, respectively. Nine protein-coding genes (*atpF, ndhA, ndhB, petB, petD, rpl2, rpl16, rpoC1*, and *rps16*) and six tRNA genes (*trnG-GCC, trnK-UUU, trnL-UAA, trnV-UAC, trnI-GAU*, and *trnA-UGC*) had a single intron, while three protein-coding genes (*ycf3, clpP*, and *rps12*) possessed two introns (Table S2). All the protein-coding genes had standard AUG as initiator codon. The gene *rps12* was found to be trans-spliced, with the 5′-end exon located in the LSC region and two copies of 3′-end exon and intron in the IR regions. The gene *ycf15* was identified as pseudogene because it contained several internal stop codons. The gene *ycf1* was located in the boundary regions between SSC/IRb. Incomplete duplication of the normal copy of *ycf1* resulted in the *ycf1* pseudogene at the SSC/IRa border in all three cp genomes (*P. subaequalis* (TX): 985 bp; *P. subaequalis* (SJD): 985 bp; *L. formosana*: 1,609 bp).

### Divergence hotspot regions of *P. subaequalis*

**Figure 3:**
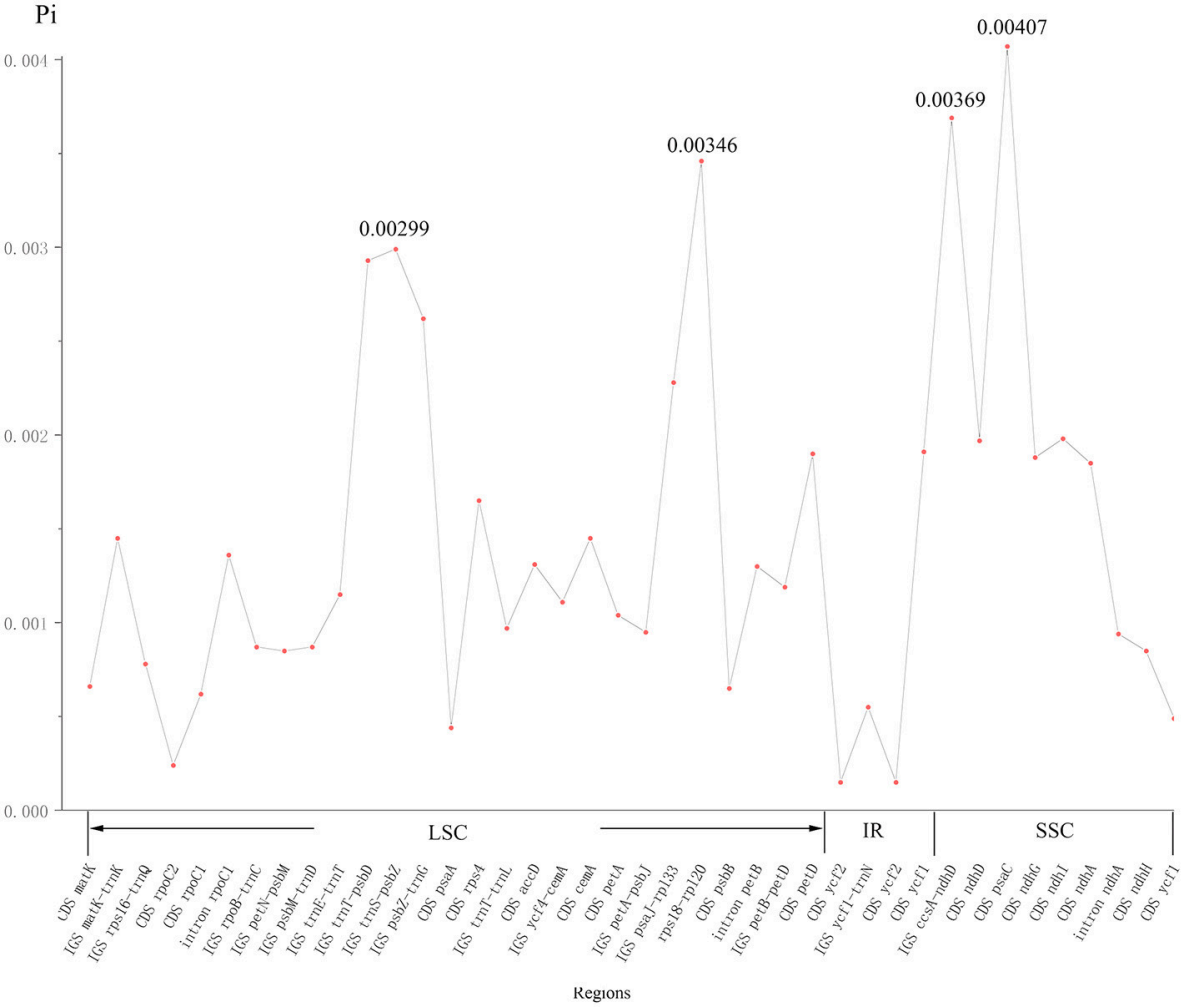
The nucleotide variability (Pi) values were compared between two *Parrotia subaequalis*.

The nucleotide variability (Pi) values in the two cp genomes of *P. subaequalis* were calculated for the 40 regions showing single-nucleotide polymorphisms (SNPs). The overall average Pi value was 0.00144. For 20 protein coding regions, Pi values ranged from 0.00015 (*ycf2*) to 0.00407 (*psaC*), among which *psaC, rps4, cemA, petD, ycf1, ndhD, ndhG, ndhI, and ndhA* regions showed remarkably higher Pi values (*Pi* > 0.00144). For the 17 intergenic spacer, *Pi*-values ranged from 0.00055 (*ycf1-trnN*) to 0.00369 (*ccsA-ndhD*), of which *matK-trnK, trnT-psbD, trnS-psbZ, psbZ-trnG, psaJ-rpl33, rps18-rpl20*, and *ccsA-ndhD* regions with Pi values exceeding the average value. Besides, for the three intron regions, *Pi*-values varied between 0.00094 (intron *ndhA*) and 0.00136 (intron *rpoC1*) (Figure 3, Table S3).

### Characterization of chloroplast SSRs and polymorphism analysis

**Figure 4:**
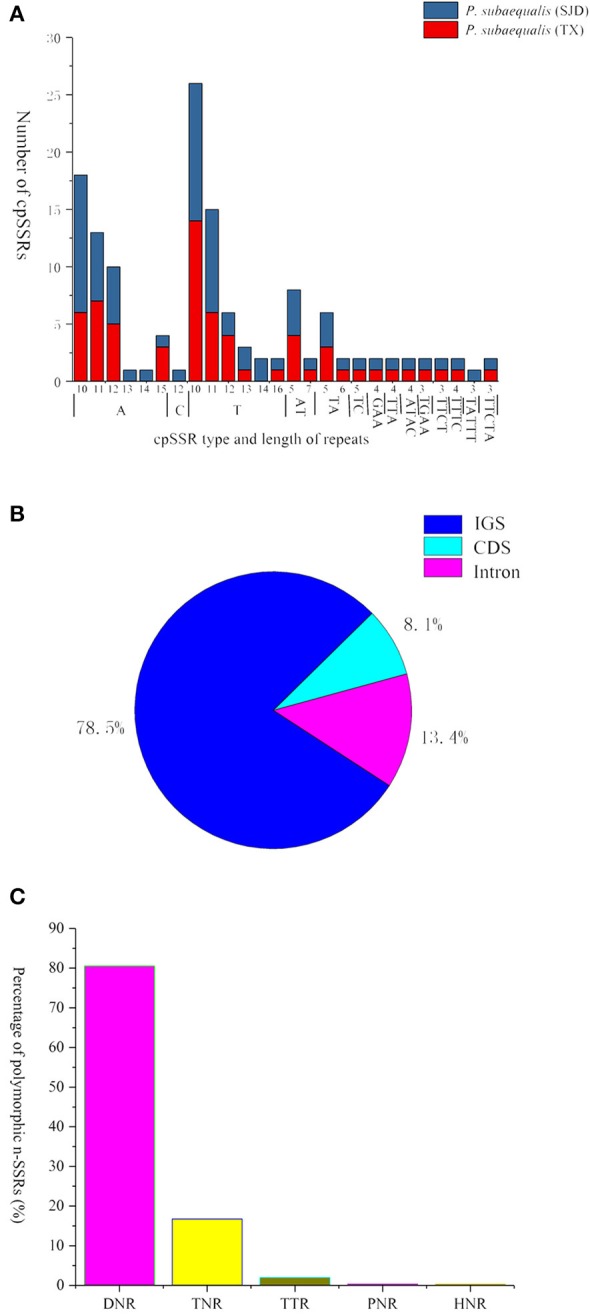
Simple sequence repeats (SSRs) in the two *Parrotia subaequalis* chloroplast genomes. **(A)** Numbers of cpSSRs type and length of repeats; **(B)** Distribution of cpSSR loci. IGS, intergenic spacer region; **(C)** Overview of the candidate polymorphic nuclear SSRs (nSSRs) detected in the two *Parrotia subaequalis*.

In total, 139 perfect cpSSRs, including 104 mononucleotide (A, C, T), 20 dinucleotide (AT, TA, TC), 4 trinucleotide (GAA, TTA), 8 tetranucleotide (ATAC, TGAA, TTCT, TTTC), and 3 pentanucleotide (TATTT, TTCTA) repeats were detected within the two *P. subaequalis* cp genomes. The mononucleotide A/T repeat units were found to be the most abundant, with 49 in *P. subaequalis* (TX) and 54 in *P. subaequalis* (SJD) (Table S4, Figure 4A). Most SSR loci were located in the IGS regions (78.5%), followed by CDS (8.1%) and introns (13.4%) (Table S4, Figure 4B). The cp genomes of *P. subaequalis* (TX) and *P. subaequalis* (SJD) contained 71 and 78 SSRs, respectively, of which 30 were potential polymorphic cpSSRs (Table S5). Based on a strict set of criteria employed in this study, 20 candidates of polymorphic SSR loci were selected from the total 30 potential polymorphic cpSSRs to evaluate their amplification and polymorphism in the initial screening with the 12 individuals from the six populations (Table S5). Finally, after excluding those that gave poor amplification or produced a complex pattern with multiple bands, 10 with high polymorphism (Table S6) were genotyped in the 96 individuals from the six populations. The 10 cpSSR loci yielded a total of 43 alleles across the 96 samples. The number of alleles (*N*_A_) per locus varied from 2 to 6, while the the unbiased haploid diversity (*H*_d_) per locus ranged from 0.392 to 0.752 (Table 3), representing the different level of genetic diversity in chloroplast genome. Moreover, all cpDNA loci were successfully amplified in all the other five species of Hamamelidaceae (Table 4).

**Table 3:** Genetic characteristics of the 10 polymorphic cpSSR loci and 12 polymorphic nSSR loci in *Parrotia subaequalis*^a^.

***Locus***	***nSSR***						***cpSSR***		
	***N*_A_**	***H*o**	***H*_E_**	***H*_S_**	***H*_T_**	**PIC**	***N*_A_**	***H*_d_**	***A*s (bp)**
PasN1/PasC2	2	0.250	0.220	0.201	0.220	0.195	6	0.628	114,115,116,118,119,120
PasN2/PasC3	2	0.073	0.071	0.067	0.071	0.068	6	0.504	228,232,233,234,236,237
PasN4/PasC5	6	0.594	0.746	0.658	0.753	0.706	4	0.512	145,149,153,154
PasN7/PasC7	14	0.990	0.868	0.740	0.865	0.849	4	0.498	178,180,182,184
PasN8/PasC9	6	0.573	0.749	0.595	0.748	0.701	5	0.573	198,199,200,206,207
PasN10/PasC11	5	0.833	0.606	0.571	0.605	0.535	2	0.392	271,276
PasN12/PasC12	6	0.635	0.634	0.563	0.633	0.602	4	0.552	271,273,274,275
PasN17/PasC13	2	0.146	0.220	0.210	0.227	0.195	3	0.479	234,235,236
PasN21/PasC16	2	0.385	0.463	0.388	0.462	0.354	4	0.587	264,266,267,268
PasN25/PasC17	6	0.542	0.730	0.550	0.726	0.687	5	0.752	189,190,191,192,193
PasN27	8	0.385	0.670	0.564	0.676	0.623			
PasN30	5	0.833	0.721	0.629	0.721	0.668			
Mean/Overall	64	0.520	0.558	0.478	0.559	0.515	43	0.548	

*N_A_, number of alleles; H_O_, observed heterozygosity for nSSR; H_E_, expected heterozygosity for nSSR; H_S_, average genetic diversity within populations for nSSR; H_T_, total genetic diversity for nSSR; H_CP_, haplotypic diversity for cpSSR; As, cpSSR allele size sampled*.

a *Voucher and locality information are provided in Table 1*.

**Table 4:** Fragment sizes detected in cross-amplification tests of the 25 newly developed microsatellite markers in the five related species of Hamamelidaceae^a^.

**Locus**	***Parrotia persica* (*n* = 3)**	***Parrotiopsis jacquemontana*(*n* = 3)**	***Sycopsis sinensis* (*n* = 3)**	***Distylium racemosum* (*n* = 3)**	***Hamamelis virginiana* (*n* = 3)**
PasN1	169	164	169	169	169
PasN2	189–196	189–196	189–196	189–196	189–196
PasN4	185–191	191–197	185–191	185–191	191–197
PasN7	–	200	–	196	–
PasN8	108–110	–	108–110	106	–
PasN10	173–179	–	173	173	173
PasN11	198	206–208	196–198	196–200	196–198
PasN12	118	114	118	118	118
PasN14	120	141	120	120	120
PasN17	196	190	181	199	181
PasN21	137	–	137	137	137
PasN24	138–147	132–141	135	138–147	135
PasN25	193	184–190	187–190	181–196	187–190
PasN27	168	168	–	156	–
PasN30	184	180–184	180–184	180–188	180–184
PasC2	119	114	116	118	116
PasC3	237	235	234	232	233
PasC5	145	153	149	149	149
PasC7	177	179	179	178	178
PasC9	199	198	199	201	199
PasC11	276	271	276	276	276
PasC12	270	272	270	269	270
PasC13	236	233	235	236	235
PasC16	264	265	263	264	264
PasC17	192	189	190	193	190

–*, amplification failed*.

a *Voucher and locality information are provided in Table 1*.

### Characterization of nuclear SSR loci and polymorphism analysis

A total of 596 *P. subaequalis* candidates of polymorphic nSSR loci with an average length of 17 bp were detected in the nuclear genome. Among these loci, di-nucleotide repeats (DNRs) were the most abundant repeat type (480; 80.54%), followed by tri- (TNRs; 100; 16.77%), tetra- (TTRs; 12; 2.01%), penta- (PNRs; 2; 0.34%) and hexa-nucleotide repeats (HNRs; 2; 0.34%) (Table S7, Figure 4C). Following a strict set of criteria, 30 candidates of polymorphic SSR loci were selected for further trials. After excluding those that gave poor amplification or produced a complex pattern with multiple bands in an initial polymorphism screening, 15 with high polymorphism (Table S8) were genotyped in the 96 individuals from the six populations. Of the 15 nSSR loci, a high frequency of null alleles was detected in PasN11 and PasN14 (*v* > 5%), while significant linkage disequilibrium was observed between PasN24 and PasN14. These three loci were excluded from subsequent analyses. Four of the remaining 12 loci deviated significantly from HWE expectations (*P* < 0.001) in some populations (PasN4 in HBS; PasN7 in ZXC and SJD; PasN10 in TX and HBS; PasN27 in LWS and WFS) as a result of heterozygote deficiency. In total, the 12 polymorphic SSRs yielded 64 alleles with an average of 5.33 alleles and a range of 2 to 14 alleles (*N*_A_) per locus. Besides, the observed (*H*_O_) and expected (*H*_E_) heterozygosity per locus over all populations ranged from 0.073 to 0.990 (with an average of 0.520) and from 0.071 to 0.868 (with an average of 0.558), respectively (Table 3). Eight nSSR loci were successfully amplified in all the other five species of Hamamelidaceae, while seven loci produced PCR fragments in some species (i.e., PasN7 for *P. persica, S. sinensis* and *H. virginiana*; PasN8 for *P. persica* and *P. jacquemontana*; PasN14 for *H. virginiana*; PasN21 and PasN10 for *P. jacquemontana*; PasN27 for *S. sinensis* and PasN25 for *H. virginiana*) (Table 4).

### Genetic diversity and population structure

For the cpSSR dataset, a total of 15 haplotypes were identified across the 96 individuals from the six populations based on the 10 polymorphic loci (H1–H15; Figure 1, Table 5). High total haplotypic diversity with *H*cp = 0.832 at species level. The haplotypic diversity per population (*H*cp) ranged from 0.520 (SJD) to 0.862 (HBS), with an average of 0.713 (Table 5). H1, H2, H3, and H4 were the most frequent haplotypes, and almost shared by all the populations. H5 and H6 were shared between LWS and ZXC, H10, H11, and H12 were shared among WFS, TX and HBS. While the remaining 6 haplotypes (40% of the total) were restricted to single populations (Figure 1, Table 5). The relationships between 15 cpSSR haplotypes were analyzed by a median-joining network model (Figure 1). Haplotype H11 occupied the central place of this network. Six median vectors (mv) representing missing intermediates were inferred from the network. The haplotype network displayed a distinct geographic distribution of haplotypes that coincided with certain mountain regions (Figure 1). For example, haplotypes H10 and H12–H14 formed one distinct clade and spanned populations (HBS, WFS and TX) in Dabie Mountain region; haplotypes H5–H9 were recovered as a separate clade and were restricted to Yellow Mountain region. The indices of population structure *G*_ST_ and *R*_ST_ were 0.335 and 0.652 respectively, whereby *R*_ST_ was significantly greater than *G*_ST_ (*P* < 0.01), indicating the existence of a marked phylogeographical structure at this scale. The AMOVA partitioned genetic variation into 65.45% among six populations and 34.55% was contributed by differences within populations (*P* < 0.001; Table S9).

**Table 5:** Genetic characteristics of the six *Parrotia subaequalis* populations studied.

***Population code***	***nSSR***				***cpSSR***			
	***N***	***A*_R_**	***H*_O_**	***H*_E_**	***N***	***N*_E_**	***H*_CP_**	***Haplotype***
SJD	16	2.122	0.510	0.431	16	1.576	0.520	H1(6), H2(4), H3(4), H4(2)
LWS	16	2.464	0.479	0.486	16	1.928	0.647	H1(2), H2(2), H4(6), H5(4), H6(2)
ZXC	16	2.007	0.552	0.453	16	2.674	0.844	H1(2), H2(2), H4(1), H5(3), H6(1), H7(3), H8(2), H9(2)
WFS	16	2.681	0.464	0.481	16	1.922	0.663	H1(4), H2(2), H3(2), H4(2), H10(4), H11(2)
TX	16	2.558	0.620	0.540	16	2.440	0.742	H1(2), H2(2), H3(2), H4(2), H10(3), H11(1), H12(4)
HBS	16	1.814	0.495	0.395	16	2.700	0.862	H1(1), H2(1), H4(1), H10(2), H12(3), H13(2), H14(3), H15(3)
Mean/Overall	96	2.274	0.520	0.464	96	2.207	0.713	H1(17), H2(13), H3(8), H4(14), H5(7), H6(3), H7(3), H8(2), H9(2), H10(9), H11(3), H12(7), H13(2), H14(3), H15(3)

*N, sample size for nSSR/cpSSR analysis; A_R_, allelic richness for nSSR; H_O_, observed heterozygosity for nSSR; H_E_, expected heterozygosity for nSSR; N_E_, number of effective alleles for cpSSR; H_CP_, haplotypic diversity*.

**Figure 5:**
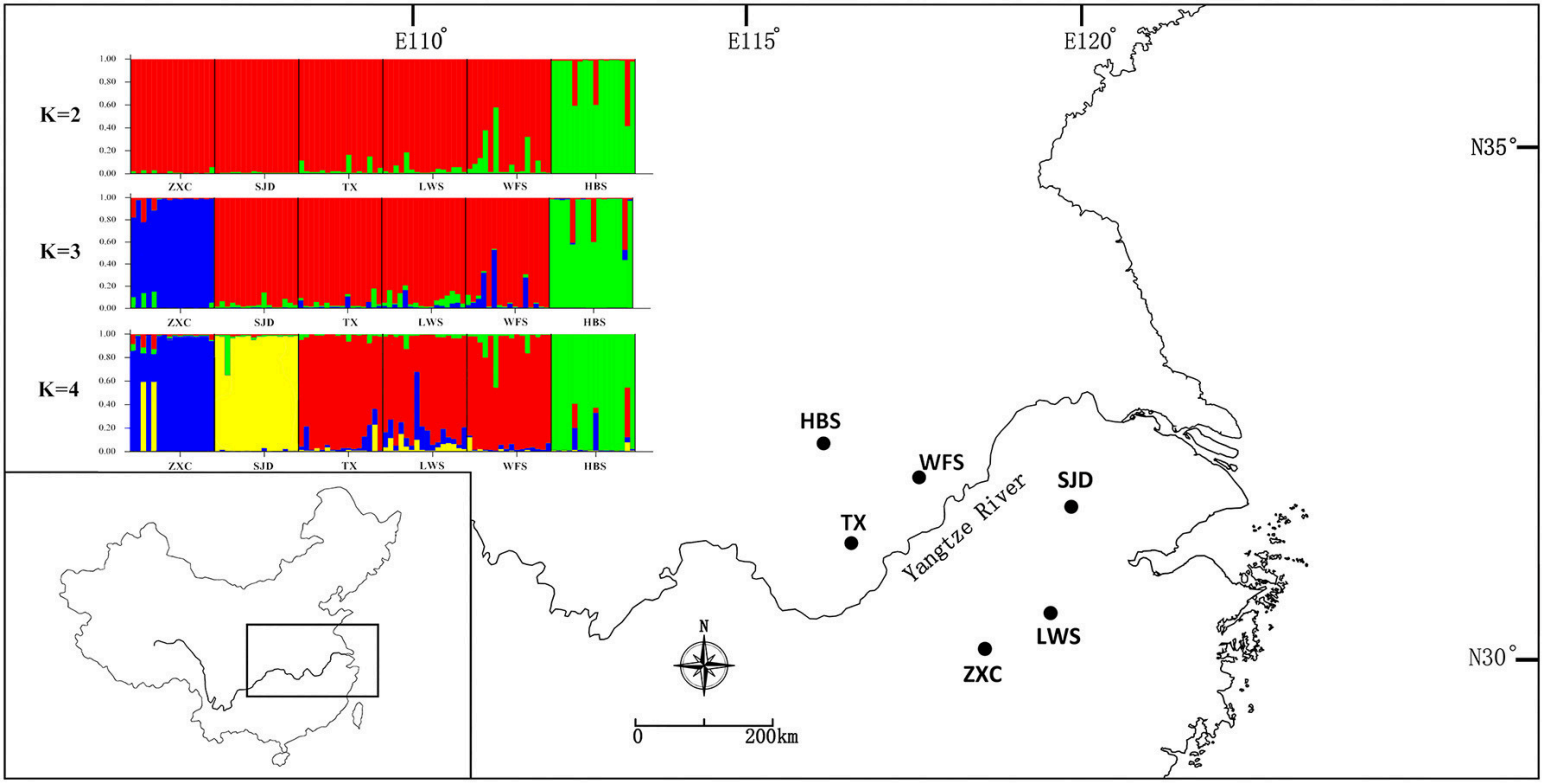
Histogram of the STRUCTURE analysis for the model with *K* = 3 (showing the highest ΔK), *K* = 2 and *K* = 4. Each color corresponds to a suggested cluster, and a vertical bar represents a single individual. The x axis corresponds to population codes. The y axis presents the estimated membership coefficient (*Q*) for each individual in the different clusters.

For the nSSR dataset, per locus estimates of the average value of genetic diversity within-populations (*H*_S_) among loci was 0.478, with a range of 0.067–0.740; while the total genetic diversity over all populations (*H*_T_) ranged from 0.071 to 0.865 and averaged 0.559 (Table 3). At the population level, average estimates of genetic diversity were generally high (*A*_R_ = 2.274, *H*_E_ = 0.464, *H*_O_ = 0.520), being lowest in population HBS (1.814, 0.395, and 0.495) and highest in population TX (2.558, 0.540, and 0.620; Table 5). The nSSR-derived data demonstrated significant population genetic differentiation at the range-wide scale (*R*_ST(n)_ = 0.331, *P* < 0.001). The hierarchical AMOVA revealed that 16.39% of the total variation was attributed to differences among six populations and that 83.61% was contributed by differences within populations (*P* < 0.001; Table S9). Mantel tests of IBD revealed no significant correlation between geographical and genetic distances (*r* = 0.228, *P* = 0.564). Using the nSSR-derived *R*_ST(n)_ value of 0.331 across the six populations surveyed, and their corresponding value for cpSSR markers, *R*_ST(cp)_ = 0.652, the pollen/seed migration ratio (*r*) was calculated as 1.78, indicating a slightly higher level of pollen flow as compared to seed flow. structure yielded the highest likelihood when samples were clustered into three groups (*K* = 3). However, all individuals were clearly assigned to non-empty groups when *K* values ranged from 2 to 4 (Figure 5). Running structure and using *K* = 2, HBS, the southernmost population, was firstly separated from the remaining 5 populations; with *K* = 3, ZXC, the westernmost population, formed a separate cluster; with *K* = 4, SJD, the easternmost population, were separated.

## Discussion

### The mining of chloroplast and nuclear SSRs

In the last decade, next-generation high-throughput sequencing technologies have allowed producing genomic resources in a rapid and cost-effective way (Mardis, 2008; Hao et al., 2011). Here, in our study, using the low-coverage whole genome shotgun sequencing data, we successfully assembled the complete chloroplast genomes of *P. subaequalis* (Figure 2). The cp genomes displayed typical quadripartite structure, which is consistent with previous published plastid genomes of four Saxifragales families (Dong et al., 2013). The genome contains 133 unique genes, 18 of which are duplicated within the IRs. Although there are expansions or contractions of IR regions observed among the cp genomes of *P. subaequalis* and the other four Saxifragales families of Saxifragales (Dong et al., 2013), they contribute little to the overall size differences in the chloroplast genomes. We found 16 regions of the two cp genomes, which were inferred to already represent polymorphic characters like in the seven intergenic spacers (*matK-trnK, trnT-psbD, trnS-psbZ, psbZ-trnG, psaJ-rpl33, rps18-rpl20*, and *ccsA-ndhD*) and the nine genes (*psaC, rps4, cemA, petD, ycf1, ndhD, ndhG, ndhI*, and *ndhA*). These regions of the cp genome can be further used to evaluate maternally inherited genomic diversity for phylogeographic and population genetic studies of *P. subaequalis*. Moreover, based on a comparative analysis of two cp genomes of *P. subaequalis*, we identified a total of 30 candidates of polymorphic cpSSR loci, for which 20 primer sets were designed to evaluate their amplification and polymorphism in the initial screening with the 12 individuals from the six populations. Of the 20 primer pairs, 10 yielded clear and polymorphic amplification products, confirming that plastome assembly from low coverage genomic sequencing is an ideal method for the development of cpSSR markers. Notably, the 10 polymorphic SSR markers could also be amplified in other five Hamamelidaceae species, indicating that they could be used as cross-species markers within Hamamelidaceae.

Genomic SSRs have traditionally been isolated by hybridizing repeat-enriched molecular probes in genomic libraries (Zalapa et al., 2008), but this method has proven low efficiency, time consuming and costly, and can isolate only the targeted enriched SSR motif types (Zane et al., 2002; Parchman et al., 2010). In addition to the chloroplast SSR loci developmenet, the present work also demonstrated that the use of sequences derived from low-coverage shotgun sequencing of genomic DNA to develop nuclear SSR markers in non-model species is a rapidly and cost-effective approach. As we used two individuals representing the northern- and southernmost distribution of *P. subaequalis* to construct the sequencing library, based on the two assembled sequences, we identified a total of 576 candidate polymorphic SSRs by means of the program CandiSSR. In agreement with previous reports from many other plant taxa (Jiao et al., 2012; Xia et al., 2016), dinucleotide motifs are found to be the most common in *P. subaequalis*. A total of 30 designed primer pairs were used for validation of the SSR markers, and 15 primer pairs (50%) were proved to be polymorphic among 96 individuals from the six populations. Such high success ratios indicate that the application of next-generation sequencing (NGS) technologies for SSRs development is possible to develop large numbers of polymorphic SSR markers. For population genetic analyses in *P. subaequalis*, 13 loci seem to provide a good resource to explore population genetic diversity and structure (see below). Our tests of interspecies transferability of the 15 SSRs from *P. subaequalis* to other five Hamamelidaceae species yielded transfer success rates of 47% (7) for all species, suggesting an intermediate level of cross-species transferability.

### Population genetic diversity and structure

Despite its restricted and highly disjunct distribution range, *P. subaequalis* exhibits similar or even higher levels of cpDNA haplotype diversity (*H*cp = 0.862) as compared to other Chinese endemic flowering plant taxa presented in previous studies by using similar markers (e.g., 0.893 for *Davidia involucrata*, Ma et al., 2015; 0.627 for *Dipteronia dyeriana*, Chen et al., 2017). The species-wide levels of haplotype diversity in *P. subaequalis* were also higher than the mean value of 170 seed plants estimated by maternally inherited markers (mean: *h*_T_ = 0.67; Petit et al., 2005). Such high total cpDNA gene diversity is generally thought to reflect the long evolutionary histories (Sun et al., 2005) but also suggests restricted gene flow between populations (Varvio et al., 1986; Qiu et al., 2009). In line with the first assumption, the estimated speciation time of around 7.5 Mya for the two extant species of *Parrotia* qualifies them as Tertiary relicts (Li and Tredici, 2008), indicating a long period of time for which both species have evolved separately in different glacial refugia. In fact, Eastern China has never been directly impacted by Pleistocene glaciations (Axelrod and Fuerch, 1996) and served as one of the most important ancient relict areas for temperate biota throughout the Quaternary (Deng et al., 1992). Therefore, *P. subaequalis* likely persisted in this long-term refuge, allowing for the maintenance of considerable genetic variation. The estimated value of pollen flow/seed flow (*r* = 1.78) is at the lower end of the range reported for 93 seed plant species (median *r* ≈ 17, Petit et al., 2005; Hodgins and Barrett, 2007). Considering the large maternal *R*_ST(cp)_ (0.652) estimated for *P. subaequalis*, its correspondingly low *r* estimate likely implies restricted pollen flow in a low-seed disperser rather than a significant role of seed flow in a species for which pollen dispersal is limited. In fact, nSSR-derived *R*_ST(n)_ value of 0.331 suggest a substantial amount of population isolation. Therefore, the reduced levels of contemporary intersite gene flow via pollen and seed agrees well with the second assumption. The above results also accord with field observations indicating that (1) the small (ca 4–5mm) seeds from the two-seeded capsules are ballistically dispersed for a short distance (≤ 18 m) (Deng et al., 1992; Yang, 1994); and (2) the wind-mediated pollen dispersal is severely constrained in *P. subaequalis*, probably as a consequence of its limitation to mountain streams and slopes as well as often steep temperate-deciduous forest habitats in isolated mountains.

In general, there is increased efficiency of genetic drift in small and isolated populations (Ellstrand and Elam, 1993; Lande, 1999). In this study, a complete absence of IBD, together with the strong population subdivision in both marker systems, suggests that genetic drift had a larger historical role in contemporary population structure and genetic diversity compared to limited pollen and seed flow alone (Hutchison and Templeton, 1999). Our nSSR-measured *H*_E_ value (*H*_E_ = 0.464) is comparable to average nSSR-based *H*_S_ (the equivalent to *H*_E_ in the present study) in endemic species (*H*_S_ = 0.42), as reviewed by Nybom (2004). Therefore, the low within-population nSSRs diversity detected in *P. subaequalis* (in terms of *H*_E_) are more likely due to the historical random loss of alleles, possibly following habitat fragmentation, rather than inbreeding. Likewise, historical processes such as long-term isolation and/or habitat fragmentation may have also contributed to the geographic differentiation of *P. subaequalis* (see also Young et al., 1996). In fact, based on structure analyses of nSSRs data, the populations HBS, ZXC (*K* = 3) and SJD (*K* = 4) were assigned to a separate cluster, respectively (Figure 5). Moreover, each of the regions Dabie Mountain and Yellow Mountain were also found to contain a set of regional-specific haplotypes (Figure 1). The spatially consistent genetic split between two marginal populations and central populations as registered by both cpSSRs and nSSRs strongly implies a long-term population persistence and isolation in eastern China, possibly before the beginning of the last glacial period. Taken together, geographical distribution patterns of haplotype (Figure 1) and nSSR cluster of *P. subaequalis* (Figure 5) suggested at least three refugia in Dabie Mountain, Yellow Mountain and central region. Nevertheless, for the central populations, it seems unlikely that ongoing gene flow among them may be existent because the smallest distances between the mountains where this species occur is 99 km (Geng et al., 2015). Thus, the cpDNA haplotypes (e.g., H1, H2, H3, H4) and nSSRs cluster (“red”) shared among populations (Figures 1, 5) may largely reflect common ancestry due to incomplete lineage sorting rather than recurrent gene flow. In fact, based on fossil-based evidence, forests were found to decline during the last 6,000 years in the middle and lower reaches of the Yellow River (Ren and Beug, 2002) and in the Yangtze delta region (Yi et al., 2003) due to the expansion of farming since the Yangshao Culture period (ca. 5,000–6,000 years ago). Such human interference may have contributed to population extinction and/or disruption of recent population connectivity of *P. subaequalis*. However, it remains to be investigated whether the separation of three clusters resulted from historical fragmentation due to human interference or eco-geographic isolation induced by Pleistocene climate change. However, irrespective of the outcome of such studies, the three genetic clusters of *P. subaequalis* revealed by both cpSSRs and nSSRs should be considered as separate conservation units in any program.

## Conclusions

To enable population genetic and phylogeographic analyses in this species, we performed paired-end shotgun sequencing (2 × 125 bp) of genomic DNA from two individuals of *P. subaequalis* on the Illumina HiSeq platform. Based on the resulting sequences, we have assembled and characterized the plastome of *P. subaequalis*. Moreover, we have identified the polymorphic cpSSRs/nSSRs using the new pipeline CandiSSR and mutational hotspots of the *P. subaequalis* plastome. The present work demonstrates the use of sequences derived from low-coverage shotgun sequencing of genomic DNA to rapidly and cost-effectively explore genomic resources in a genetically understudied plants.

We further used 10 polymorphic cpSSR and 12 polymorphic nSSR loci to investigate the genetic diversity and population structure for six populations sampled across the range of *P. subaequalis*. Our results revealed that *P. subaequalis* exhibits abundant genetic diversity (e.g., cpSSRs: *H*cp = 0.832; *H*_T_ = 0.559) and high genetic differentiation; e.g., cpSSRs: *R*_ST(cp)_ = 0.652; nSSRs: *R*_ST(n)_ = 0.331], and is characterized by a low pollen-to-seed migration ratio (*r* ≈ 1.78). These genetic patterns are attributable to its long evolutionary histories and reduced levels of contemporary intersite gene flow via pollen and seed. In addition, lack of isolation-by-distance pattern, together with the strong population subdivision in both marker systems, suggests that long-term isolation, and/or habitat fragmentation as well as genetic drift may have also contributed to the geographic differentiation of *P. subaequalis*. Therefore, long-term habitat protection is the most important means to prevent further losses of genetic variation and a decrease in effective population size. Moreover, the three genetic clusters of *P. subaequalis* revealed by both cpSSRs and nSSRs should be considered as separate conservation units in any program, allowing for the long-term preservation of its genetic resources.

## Author contributions

Y-YZ, Y-XQ, and Z-SW conceived the study; Y-YZ analyzed the data and wrote the manuscript; ES and Z-PY helped to collect samples; Y-YZ and ES performed the experiment; Z-PY and Q-FG provided useful suggestions on the manuscript.

### Conflict of interest statement

The authors declare that the research was conducted in the absence of any commercial or financial relationships that could be construed as a potential conflict of interest.
