# Supplementary material for: Development and Application of Genomic Resources in an Endangered Palaeoendemic Tree, Parrotia subaequalis (Hamamelidaceae) From Eastern China
Source: Front Plant Sci. 2018 Mar 1;9:246. doi: 10.3389/fpls.2018.00246 (PMC5838013; doi:10.3389/fpls.2018.00246)
Supplement: Supplementary file 1 [file Table1.DOCX]

**Table S1. List of all pairs of primers used to confirm accuracy of the four junctions between LSC/IRs and SSC/IRs of the two chloroplast genomes of *Parrotia subeaqualis*.**

| Regions | Sequence (5’>3’) |
| --- | --- |
| Junction of LSC/IRb | F GTATGGCCGATCATTGTG |
|  | R CTTCGTCGCCGTAGTAAA |
| Junction of IRb/SSC | F TTCGGAAGAAAAGGAGGA |
|  | R GAATTTGAATTAAACCAG |
| Junction of SSC/IRa | F TATAGTCGAAAAGAAGTGTT |
|  | R AGTGAATGGAAAGGAAAA |
| Junction of IRa/LSC | F CTTCGTCGCCGTAGTAAA |
|  | R AAGGCAGTGGATTGTGAA |
