# Supplementary material for: Development and Application of Genomic Resources in an Endangered Palaeoendemic Tree, Parrotia subaequalis (Hamamelidaceae) From Eastern China
Source: Front Plant Sci. 2018 Mar 1;9:246. doi: 10.3389/fpls.2018.00246 (PMC5838013; doi:10.3389/fpls.2018.00246)
Supplement: Supplementary file 2 [file Table2.DOCX]

**Table S2. Gene composition in two *Parrotia subaequalis* chloroplast genomes.**

| **Groups of genes** | **Names of genes** |
| --- | --- |
| Ribosomal RNAs | *rrn16*(×2), *rrn23*(×2)*, rrn4.5*(×2)*, rrn5*(×2) |
| Transfer RNAs | *trnH-GUG, trnK-UUU^a^, trnQ-UUG, trnS-GCU, trnG-GCC^a^, trnG-UCC, trnR-UCU, trnC-GCA, trnD-GUC, trnY-GUA, trnE-UUC, trnT-GGU, trnS-UGA, trnfM-CAU, trnS-GGA, trnT-UGU, trnL-UAA^a^, trnF-GAA, trnV-UAC^a^, trnM-CAU, trnW-CCA, trnP-UGG, trnI-CAU*(×2), *trnL-CAA*(×2), *trnV-GAC*(×2)*, trnI-GAU^a^*(×2), *trnA-UGC^a^*(×2), *trnR-ACG*(×2), *trnN-GUU*(×2)*, trnL-UAG* |
| Photosystem I | *psaB, psaA, psaI, psaJ, psaC* |
| Photosystem II | *psbA, psbK, psbI, psbM, psbD, psbC, psbZ, psbJ, psbL, psbF, psbE, psbB, psbT, psbN, psbH* |
| Cytochrome | *petN, petA, petL, petG, petB^a^, petD^a^* |
| ATP synthase | *atpA, atpF^a^, atpH, atpI, atpE, atpB* |
| Rubisco | *rbcL* |
| NADH dehydrogenease | *ndhJ, ndhK, ndhC, ndhB^a^*(×2), *ndhF, ndhD, ndhE, ndhI, ndhA^a^, ndhH* |
| ATP-dependent protease subunit P | *clpP^b^* |
| Chloroplast envelop membrane protein | *cemA* |
| Large units | *rpl33, rpl20, rpl36, rpl14, rpl16^a^, rpl22, rpl2^a^*(×2)*, rpl23*(×2)*, rpl32* |
| Small units | *rps16^a^, rps2, rps14, rps4, rps18, rps12^b^*(×2)*, rps11, rps8, rps3, rps19, rps7*(×2)*, rps15* |
| RNA polymerase | *rpoC2, rpoC1^a^, rpoB, rpoA* |
| Translational initiation factor | *infA* |
| Miscellaneous proteins | *matK, accD, ccsA* |
| Hypothetical proteins & Conserved reading frame | *ycf3^b^, ycf4, ycf2*(×2)*, ycf1* |
| Pseudogene | ^ψ^*ycf1,* ^ψ^*ycf15*(×2) |

^a^ Indicates the genes containing a single intron.

^b^ Indicates the genes containing two introns.

(×2) indicates genes duplicated in the IR regions.

Pseudogene is represented by ψ.
