# Supplementary material for: Development and Application of Genomic Resources in an Endangered Palaeoendemic Tree, Parrotia subaequalis (Hamamelidaceae) From Eastern China
Source: Front Plant Sci. 2018 Mar 1;9:246. doi: 10.3389/fpls.2018.00246 (PMC5838013; doi:10.3389/fpls.2018.00246)
Supplement: Supplementary file 3 [file Table3.DOCX]

**Table S3. Nucleotide variability (Pi) values and total number of mutation (Eta) in *Parrotia subaequalis.***

| Name | Length | Eta | Pi | Region |
| --- | --- | --- | --- | --- |
| CDS matK | 1515 | 1 | 0.00066 | LSC |
| IGS matK-trnK | 690 | 1 | 0.00145 |  |
| IGS rps16-trnQ | 2588 | 2 | 0.00078 |  |
| CDS rpoC2 | 4164 | 1 | 0.00024 |  |
| CDS rpoC1 | 1625 | 1 | 0.00062 |  |
| intron rpoC1 | 736 | 1 | 0.00136 |  |
| IGS rpoB-trnC | 1149 | 1 | 0.00087 |  |
| IGS petN-psbM | 1181 | 1 | 0.00085 |  |
| IGS psbM-trnD | 1156 | 1 | 0.00087 |  |
| IGS trnE-trnT | 872 | 1 | 0.00115 |  |
| IGS trnT-psbD | 1369 | 4 | 0.00293 |  |
| IGS trnS-psbZ | 340 | 1 | 0.00299 |  |
| IGS psbZ-trnG | 396 | 1 | 0.00262 |  |
| CDS psaA | 2253 | 1 | 0.00044 |  |
| CDS rps4 | 606 | 1 | 0.00165 |  |
| IGS trnT-trnL | 1028 | 1 | 0.00097 |  |
| CDS accD | 1530 | 2 | 0.00131 |  |
| IGS ycf4-cemA | 902 | 1 | 0.00111 |  |
| CDS cemA | 690 | 1 | 0.00145 |  |
| CDS petA | 963 | 1 | 0.00104 |  |
| IGS petA-psbJ | 1054 | 1 | 0.00095 |  |
| IGS psaJ-rpl33 | 439 | 1 | 0.00228 |  |
| IGS rps18-rpl20 | 289 | 1 | 0.00346 |  |
| CDS psbB | 1527 | 1 | 0.00065 |  |
| intron petB | 772 | 1 | 0.0013 |  |
| IGS petB-petD | 841 | 1 | 0.00119 |  |
| CDS petD | 525 | 1 | 0.0019 |  |
| CDS ycf2 | 6879 | 1 | 0.00015 | IR |
| IGS ycf1-trnN | 1830 | 1 | 0.00055 |  |
| CDS ycf2 | 6879 | 1 | 0.00015 |  |
| CDS ycf1 | 1049 | 2 | 0.00191 |  |
| IGS ccsA-ndhD | 271 | 1 | 0.00369 | SSC |
| CDS ndhD | 1521 | 3 | 0.00197 |  |
| CDS psaC | 246 | 1 | 0.00407 |  |
| CDS ndhG | 531 | 1 | 0.00188 |  |
| CDS ndhI | 504 | 1 | 0.00198 |  |
| CDS ndhA | 540 | 1 | 0.00185 |  |
| intron ndhA | 1064 | 1 | 0.00094 |  |
| CDS ndhH | 1182 | 1 | 0.00085 |  |
| CDS ycf1 | 4113 | 2 | 0.00049 |  |
