# Supplementary material for: Development and Application of Genomic Resources in an Endangered Palaeoendemic Tree, Parrotia subaequalis (Hamamelidaceae) From Eastern China
Source: Front Plant Sci. 2018 Mar 1;9:246. doi: 10.3389/fpls.2018.00246 (PMC5838013; doi:10.3389/fpls.2018.00246)
Supplement: Supplementary file 4 [file Table4.DOCX]

**Table S4. The simple sequence repeats in two *Parrotia subaequalis* chloroplast genomes.**

| P. subaequalis (TX) | | | | | P. subaequalis (SJD) | | | | |
| --- | --- | --- | --- | --- | --- | --- | --- | --- | --- |
| Repeat  unit | No.repeat  unit | Start  position | End  position | Location | Repeat  unit | No.repeat  unit | Start  position | End  position | Location |
| A | 10 | 3850 | 3859 | IGS (matK-trnK) | A | 10 | 3850 | 3859 | IGS (matK-trnK) |
|  |  | 4770 | 4779 | IGS (trnK-rps16) |  |  | 9738 | 9747 | IGS (trnS-trnG) |
|  |  | 16007 | 16016 | IGS (atpH-atpI) |  |  | 16014 | 16023 | IGS (atpH-atpI) |
|  |  | 32301 | 32310 | IGS (psbM-trnD) |  |  | 23974 | 23983 | Intron (rpoC1) |
|  |  | 32792 | 32801 | IGS (psbM-trnD) |  |  | 31148 | 31157 | IGS (petN-psbM) |
|  |  | 50464 | 50473 | Intron (trnL) |  |  | 32308 | 32317 | IGS (psbM-trnD) |
|  | 11 | 23 | 33 | IGS (trnH-GUG) |  |  | 32799 | 32808 | IGS (psbM-trnD) |
|  |  | 29402 | 29412 | IGS (rpoB-trnC) |  |  | 35409 | 35418 | IGS (trnT-psbD) |
|  |  | 35391 | 35401 | IGS (trnT-psbD) |  |  | 60512 | 60521 | IGS (rbcL-accD) |
|  |  | 47736 | 47746 | IGS (ycf3-trnS) |  |  | 67382 | 67391 | IGS (petA-psbJ) |
|  |  | 73966 | 73976 | Intron (clpP) |  |  | 84759 | 84768 | IGS (rpl14-rpl16) |
|  |  | 140368 | 140378 | Intron (trnI) |  |  | 123741 | 123750 | IGS (ndhG-ndhL) |
|  |  | 159258 | 159268 | CDS (rpl2) |  | 11 | 4770 | 4780 | IGS (trnK-rps16) |
|  | 12 | 382 | 393 | IGS (trnH-psbA) |  |  | 29410 | 29420 | IGS (rpoB-trnC) |
|  |  | 9732 | 9743 | IGS(trnS-trnG) |  |  | 47770 | 47780 | IGS (ycf3-trnS) |
|  |  | 14616 | 14627 | IGS(atpF-atpH) |  |  | 50498 | 50508 | Intron (trnL) |
|  |  | 23974 | 23985 | LSC rpoC1 intron |  |  | 117915 | 117925 | IGS (rpl32-trnL) |
|  |  | 31139 | 31150 | IGS(petN-psbM) |  |  | 140411 | 140421 | Intron (trnI) |
|  |  | 35272 | 35283 | IGS(trnT-psbD) |  | 12 | 22 | 33 | IGS (trnH-GUG) |
|  |  | 74904 | 74915 | LSC ClpP intron |  |  | 382 | 393 | IGS (trnH-psbA) |
|  | 15 | 12869 | 12883 | IGS (atpA-atpF) |  |  | 35290 | 35301 | IGS (trnT-psbD) |
|  |  | 40462 | 40476 | IGS (trnfM-psaB) |  |  | 74941 | 74952 | Intron (clpP) |
|  |  | 47475 | 47489 | Intron (ycf3) |  |  | 159301 | 159312 | IGS (rpl2) |
| T | 10 | 5184 | 5193 | IGS (trnK-rps16) |  | 13 | 40499 | 40511 | IGS (rps14-psaB) |
|  |  | 7759 | 7768 | IGS (rps16-trnQ) |  | 14 | 47510 | 47523 | Intron (ycf3) |
|  |  | 10400 | 10409 | IGS (trnS-trnG) |  | 15 | 12883 | 12897 | IGS (atpA-atpF) |
|  |  | 10761 | 10770 | IGS (trnS-trnG) | C | 12 | 5456 | 5467 | IGS (rps16-trnQ) |
|  |  | 14326 | 14335 | IGS (atpF-atpH) | T | 10 | 5185 | 5194 | IGS (trnK-rps16) |
|  |  | 15866 | 15875 | IGS (atpH-atpI) |  |  | 6502 | 6511 | IGS (rps16-trnQ) |
|  |  | 27645 | 27654 | CDS (rpoB) |  |  | 7770 | 7779 | IGS (rps16-trnQ) |
|  |  | 38444 | 38453 | IGS (psbC-trnS) |  |  | 14337 | 14346 | IGS (atpF-atpH) |
|  |  | 39783 | 39792 | IGS (trnG-trnfM) |  |  | 15873 | 15882 | IGS (atpH-atpI) |
|  |  | 51515 | 51524 | IGS (trnF-ndhJ) |  |  | 27652 | 27661 | CDS (rpoC2) |
|  |  | 57802 | 57811 | CDS (atpB) |  |  | 39821 | 39830 | IGS (trnG-trnfM) |
|  |  | 72746 | 72755 | IGS (rpl20-rps12) |  |  | 57839 | 57848 | CDS (atpB) |
|  |  | 74246 | 74255 | Intron (clpP) |  |  | 72784 | 72793 | IGS (rpl20-rps12) |
|  |  | 83708 | 83717 | IGS (infA-rps8) |  |  | 83750 | 83759 | IGS (infA-rps8) |
|  | 11 | 5592 | 5602 | IGS (rps16-trnQ) |  |  | 117232 | 117241 | IGS (ndhF-rpl32) |
|  |  | 67248 | 67258 | IGS (petA-psbJ) |  |  | 118963 | 118972 | IGS (trnL-ccsA) |
|  |  | 75265 | 75275 | Intron (clpP) |  | 11 | 10412 | 10422 | IGS (trnS-trnG) |
|  |  | 87936 | 87946 | IGS (rps19-rpl2) |  |  | 10774 | 10784 | IGS (trnS-trnG) |
|  |  | 106826 | 106836 | Intron (trnI) |  |  | 17712 | 17722 | IGS (rps2-rpoC2) |
|  |  | 118919 | 118929 | IGS (trnL-ccsA) |  |  | 38461 | 38471 | IGS (psbC-trnS) |
|  | 12 | 17705 | 17716 | CDS (rps2) |  |  | 51550 | 51560 | IGS (trnF-ndhJ) |
|  |  | 51890 | 51901 | IGS (trnF-ndhJ) |  |  | 74282 | 74292 | Intron (clpP) |
|  |  | 81514 | 81525 | IGS (petD-rpoA) |  |  | 81552 | 81562 | IGS (petD-rpoA) |
|  |  | 118274 | 118285 | IGS (rpl32-trnL) |  |  | 106868 | 106878 | Intron (trnI) |
|  | 13 | 19946 | 19958 | CDS (rpoC2) |  |  | 118319 | 118329 | IGS (rpl32-trnL) |
|  | 16 | 51698 | 51713 | IGS (trnF-ndhJ) |  | 12 | 75302 | 75313 | Intron (clpP) |
| AT | 5 | 21325 | 21334 | CDS (rpoC2) |  |  | 87977 | 87988 | IGS (rps19-rpl2) |
|  |  | 45434 | 45443 | IGS (psaA-ycf3) |  | 13 | 19952 | 19964 | CDS (rpoC2) |
|  |  | 62633 | 62642 | IGS (accD-psaI) |  |  | 51926 | 51938 | IGS (trnF-ndhJ) |
|  |  | 148993 | 149002 | IGS (ndhB-trnL) |  | 14 | 5598 | 5611 | IGS (rps16-trnQ) |
|  | 7 | 121734 | 121747 | IGS (ndhD-psaC) |  |  | 120103 | 120116 | IGS (ccsA-ndhD) |
| TA | 5 | 33924 | 33933 | IGS (trnE-trnT) |  | 16 | 51734 | 51749 | IGS (trnF-ndhJ) |
|  |  | 71511 | 71520 | IGS (rpl33-rps18) | AT | 5 | 21331 | 21340 | CDS (rpoC2) |
|  |  | 98201 | 98210 | IGS (trnL-ndhB) |  |  | 45469 | 45478 | IGS (psaA-ycf3) |
|  | 6 | 48978 | 48989 | IGS (rps4-trnT) |  |  | 62671 | 62680 | IGS (accD-psaI) |
| TC | 5 | 64915 | 64924 | CDS (cemA) |  |  | 149036 | 149045 | IGS (ndhB-trnL) |
| GAA | 4 | 72070 | 72081 | IGS (rpl18-rpl20) |  | 7 | 121778 | 121791 | IGS (ndhD-psaC) |
| TTA | 4 | 49555 | 49566 | IGS (trnT-trnL) | TA | 5 | 33939 | 33948 | IGS (trnE-trnT) |
| ATAC | 4 | 7051 | 7066 | IGS (rps16-trnQ) |  |  | 71549 | 71558 | IGS (rpl33-rps18) |
| TGAA | 3 | 1755 | 1766 | IGS (psbA-matK) |  |  | 98243 | 98252 | IGS (trnL-ndhB) |
| TTCT | 3 | 32012 | 32023 | IGS (psbM-trnD) |  | 6 | 49012 | 49023 | IGS (rps4-trnT) |
| TTTC | 4 | 68751 | 68766 | IGS (psbE-petL) | TC | 5 | 64953 | 64962 | CDS (cemA) |
| TTCTA | 3 | 67002 | 67016 | IGS (petA-psbJ) | GAA | 4 | 72108 | 72119 | IGS (rpl18-rpl20) |
|  |  |  |  |  | TTA | 4 | 49589 | 49600 | IGS (trnT-trnL) |
|  |  |  |  |  | ATAC | 4 | 7061 | 7076 | IGS (rps16-trnQ) |
|  |  |  |  |  | TGAA | 3 | 1755 | 1766 | IGS (psbA-matK) |
|  |  |  |  |  | TTCT | 3 | 32019 | 32030 | IGS (psbM-trnD) |
|  |  |  |  |  | TTTC | 4 | 68789 | 68804 | IGS (psbE-petL) |
|  |  |  |  |  | TATTT | 3 | 38687 | 38701 | IGS (trnS-psbZ) |
|  |  |  |  |  | TTCTA | 3 | 67040 | 67054 | IGS (petA-psbJ) |
