# Supplementary material for: Development and Application of Genomic Resources in an Endangered Palaeoendemic Tree, Parrotia subaequalis (Hamamelidaceae) From Eastern China
Source: Front Plant Sci. 2018 Mar 1;9:246. doi: 10.3389/fpls.2018.00246 (PMC5838013; doi:10.3389/fpls.2018.00246)
Supplement: Supplementary file 6 [file Table6.DOCX]

**Table S6. Characteristics of the 10 newly developed chloroplast microsatellite loci in *Parrotia subaequalis.*^a^**

| Locus | Primer sequences (5’-3’) | Repeat motif | Allele size range (bp)^b^ | Fluorescent dye^c^ | Position | GenBank accession no. |
| --- | --- | --- | --- | --- | --- | --- |
| PasC2 | F: TCTCTTCGGGATCGAACATC | (C)_12_ | 114-120 | TAMRA | *rps16/trnQ* | MG827192 |
|  | R: GCCGTACGAGGAGAAAACCT |  |  |  |  |  |
| PasC3 | F: CTCGTACGGCTCGAGAAAAA | (T)_14_ | 228-237 | ROX | *rps16/trnQ* | MG827193 |
|  | R: AAGGGGGTTAGAGACCACTCA |  |  |  |  |  |
| PasC5 | F: TCGAAACACCGTTGTCACTG | (ATAC)_4_ | 149-153 | HEX | *rps16/trnQ* | MG827194 |
|  | R: AGGCAGGGGGTGATTTAGAC |  |  |  |  |  |
| PasC7 | F: AGAATTCGAAAAAGCAAGCG | (A)_12_ | 178-182 | TAMRA | *atpF/atpH* | MG827195 |
|  | R: CGGAGGGAAAAATACGAGGT |  |  |  |  |  |
| PasC9 | F: TTGAATCATCTGTCAACGGC | (A)_10_ | 198-207 | FAM | *petN/psbM* | MG827196 |
|  | R: TCGAGAGGCAACTCTTACGG |  |  |  |  |  |
| PasC11 | F: ATAGCCGAGCCATTCCCTAT | (TATTT)_3_ | 271-276 | TAMRA | *trnS/psbZ* | MG827197 |
|  | R: CAGATAGATCGACCCAGGGA |  |  |  |  |  |
| PasC12 | F: TTGCATCCTAAGCTCGTGACA | (A)_13_ | 271-275 | HEX | *rps14/psaB* | MG827198 |
|  | R: ACTGGTGATGAGGTGGCAAG |  |  |  |  |  |
| PasC13 | F: TTGAGCAAATAGAACGTCTTCA | (A)_10_ | 234-236 | FAM | *petA/psbJ* | MG827199 |
|  | R: CCGAACATCTGTTCCTCGAT |  |  |  |  |  |
| PasC16 | F: TCCGCTACATTCAAATGGGT | (T)_11_ | 266-267 | TAMRA | *rpl14/rpl16* | MG827200 |
|  | R: TATGAAATGGGCGGAGTAGC |  |  |  |  |  |
| PasC17 | F: TGAAATGGAACTCTCCTCGC | (A)_11_ | 189-192 | ROX | *rpl32/trnL* | MG827201 |
|  | R: GTAACAAATGGGTCGATGGG |  |  |  |  |  |

^a^The annealing temperature for all loci was 55°C.

^b^Size range values based on 96 individuals.

^c^Forward 5’label.
