# Supplementary material for: Development and Application of Genomic Resources in an Endangered Palaeoendemic Tree, Parrotia subaequalis (Hamamelidaceae) From Eastern China
Source: Front Plant Sci. 2018 Mar 1;9:246. doi: 10.3389/fpls.2018.00246 (PMC5838013; doi:10.3389/fpls.2018.00246)
Supplement: Supplementary file 8 [file Table8.DOCX]

**Table S8. Characteristics of 15 newly developed nuclear microsatellite loci in *Parrotia subaequalis.***

| Locus | Primer sequences (5’-3’) | Repeat motif | Allele size range (bp)^a^ | Flourescent dye^b^ | Ta(°C) | GenBank accession no. |
| --- | --- | --- | --- | --- | --- | --- |
| PasN1 | F:GGCATGCTGTTACTTTTCTAGCC | (AAAAG)_5_ | 159-169 | FAM | 56 | MG827202 |
|  | R: AGGTGCTCCAGCTCAACTATG |  |  |  |  |  |
| PasN2 | F: TGCCCCTAAGCAAAATGAGC | (AAAT)_5_ | 189-197 | FAM | 56 | MG827203 |
|  | R: AGAGTGCAGACGTGGAGTTG |  |  |  |  |  |
| PasN4 | F: ACATGACACACCAACTACACT | (AAG)_11_ | 185-200 | HEX | 56 | MG827204 |
|  | R: TCCTCGTCCTCGTCTTCTTCA |  |  |  |  |  |
| PasN7 | F: ACTGGGAAAGACTAAATGCA | (AG)_9_ | 188-212 | TAMRA | 55 | MG827205 |
|  | R: TCCGTAGCAGTGTCTTCCATC |  |  |  |  |  |
| PasN8 | F: ACTACCAAATTCCCACCAGCA | (AG)_9_ | 106-114 | ROX | 55 | MG827206 |
|  | R: TGTGGGCTTTACTTTCGTTGC |  |  |  |  |  |
| PasN10 | F: TCTTCCTGGTGCTATGCTTCA | (AGC)_6_ | 170-189 | HEX | 56 | MG827207 |
|  | R:GTTCAAAATCGGGCTAGAGCAG |  |  |  |  |  |
| PasN11 | F: TTGCATCCTAAGCTCGTGACA | (AT)_7_ | 194-208 | HEX | 55 | MG827208 |
|  | R: ACTGGTGATGAGGTGGCAAG |  |  |  |  |  |
| PasN12 | F: GTTGTTGTTTTGTTGGCCTA | (ATAC)_7_ | 110-139 | HEX | 56 | MG827209 |
|  | R: GCAGCAGTTCCAATGGAAGTC |  |  |  |  |  |
| PasN14 | F: TGGTGGTTGACAAGGATGCA | (CAT)_5_ | 120-132 | ROX | 56 | MG827210 |
|  | R: CTCATCGCTAAGTCTACCAGCA |  |  |  |  |  |
| PasN17 | F: CTTACACAGGCAGCTCGACA | (CTT)_6_ | 193-196 | HEX | 56 | MG827211 |
|  | R: CTGCCTCGAGACACTTCAGT |  |  |  |  |  |
| PasN21 | F: ACTTCTCTTTCGAATTGGGT | (GAT)_5_ | 131-137 | HEX | 56 | MG827212 |
|  | R: ACTGTAACACATTACTCACCAC |  |  |  |  |  |
| PasN24 | F: AAGCAAACATGGCCCAGACT | (TTC)_12_ | 132-153 | TAMRA | 56 | MG827213 |
|  | R: CTTGTACCTGTTCGGCCCAT |  |  |  |  |  |
| PasN25 | F: TTGTGTGAAGTTTGCTCGGT | (TTC)_6_ | 184-205 | ROX | 56 | MG827214 |
|  | R: ACTTGCATCCCAACACCACT |  |  |  |  |  |
| PasN27 | F: CAACGGTGGTATCAGAGCCA | (TTTA)_5_ | 156-172 | ROX | 56 | MG827215 |
|  | R: ACCATTGTGCTCCTCGTCTC |  |  |  |  |  |
| PasN30 | F: TCCTCAGCTGTTCAAACTCTCA | (TTTC)_7_ | 176-192 | FAM | 56 | MG827216 |
|  | R: ATTTACCGGCGTGAACCCTT |  |  |  |  |  |

^a^Size range values based on 96 individuals.

^b^Forward 5’label.
