# Supplementary material for: Development and Application of Genomic Resources in an Endangered Palaeoendemic Tree, Parrotia subaequalis (Hamamelidaceae) From Eastern China
Source: Front Plant Sci. 2018 Mar 1;9:246. doi: 10.3389/fpls.2018.00246 (PMC5838013; doi:10.3389/fpls.2018.00246)
Supplement: Supplementary file 9 [file Table9.DOCX]

**Table S9. Analysis of molecular variance (AMOVA) within/among the six *Parrotia subaequalis* populations using nSSRs and cpSSRs data.**

| SSR types | Source of variation | *d*_f_ | SSD | Variance components | Percentage variation | *P*-value |
| --- | --- | --- | --- | --- | --- | --- |
| nSSR | Among populations | 5 | 104.573 | 0.56371 | 16.39% | < 0.001 |
|  | Within populations | 186 | 534.938 | 2.87601 | 83.61% | < 0.001 |
| cpSSR | Among populations | 5 | 394.656 | 0.23878 | 65.45% | < 0.001 |
|  | Within populations | 90 | 12.603 | 0.12603 | 34.55% | < 0.001 |

*Note*: *d*_f_ = degrees of freedom; SSD = sum of squared deviation; *P*-value = probability.
